# High satisfaction and functional improvement following robotic‐assisted total knee arthroplasty: A Latin American cohort study

**DOI:** 10.1002/jeo2.70344

**Published:** 2025-07-07

**Authors:** Rafael Calvo Rodríguez, Waldo Gonzalez Duque, David Figueroa Poblete, Jorge Isla Villanueva, Daniela Landea Caroca, Loreto Figueroa Berrios

**Affiliations:** ^1^ Department of Traumatology, Knee and Arthroscopy Unit, Clinica Alemana Universidad del Desarrollo Santiago Chile; ^2^ Hospital del Salvador Santiago Chile; ^3^ Service of Physical Medicine and Rehabilitation, Clinica Alemana Santiago Chile

**Keywords:** outcomes, patient satisfaction, robotic assisted, total knee arthroplasty

## Abstract

**Purpose:**

Total knee arthroplasty (TKA) is a widely performed surgical procedure for patients with severe knee osteoarthritis, aiming to reduce pain, improve function, and enhance quality of life. Patient satisfaction following TKA typically ranges from 85% to 90%, with factors such as malalignment and postoperative instability being common causes of dissatisfaction. Robotic‐assisted TKA (RA‐TKA) has demonstrated advantages in surgical precision and better functional recovery compared to conventional techniques. The objective of our study is to evaluate and quantify the level of patient satisfaction and functional outcomes after robotic‐assisted TKA.

**Methods:**

Prospective cohort of patients who underwent TKA using robotic‐assisted surgery at a single centre between 2018 and 2020. Demographic data were collected, and patient satisfaction was evaluated at the 1‐year follow‐up using the Knee Society Scoring (KSS) system. Functionality was assessed through patient‐reported outcome measures (PROMs), specifically the Knee Injury and Osteoarthritis Outcome Score for Joint Replacement (KOOS‐JR), both preoperatively and postoperatively, with a minimum follow‐up period of 3 years. All statistical analyses were performed using STATA version 18.5.

**Results:**

A total of 270 patients with complete follow‐up were evaluated. 92.6% (*n* = 250) of patients were satisfied or very satisfied with the surgery, while 7.4% (*n* = 20) reported dissatisfaction. No statistically significant difference was found in age, sex, BMI, or preoperative Knee Injury and Osteoarthritis Outcome Score for Joint Replacement (KOOS JR) between the satisfaction and dissatisfaction groups. However, satisfied patients had a significantly higher postoperative KOOS JR (*p* = 0.0001).

**Conclusion:**

A high level of satisfaction and significant functional improvements were achieved after robotic‐assisted TKA in patients with severe osteoarthritis.

**Level of Evidence:**

Level II.

**Abbreviations**
BMI: body mass index
CT: computed tomography
HADS: Hospital Anxiety and Depression Scale
HKA: hip‐knee‐ankle
KOOS‐JR: Knee Injury and Osteoarthritis Outcome Score for Joint Replacement
KSS: Knee Society Scoring
MPTA: medial proximal tibial angle
PCL: posterior cruciate ligament
PROM: patient‐reported outcome measures
RA‐TKA: robotic‐assisted TKA
TKA: total knee arthroplasty

## INTRODUCTION

Total knee arthroplasty (TKA) is a common surgical procedure performed on patients with severe knee osteoarthritis, primarily aimed at reducing pain, restoring function, and improving quality of life. Between 2017 and 2019 alone, 312.167 primary knee replacements were recorded in the United Kingdom, representing 24% of the national registry [3]. In the United States, it is projected that by 2030, TKA surgeries will increase by 673%, reaching 3.48 million procedures annually due to an aging population and the rising incidence of osteoarthritis [14].

Patient satisfaction following TKA typically ranges from 80% to 90% [8, 11, 19]. This variability prompts us to delve deeper into the causes of patient discomfort and seek solutions. Several factors contribute to dissatisfaction following TKA, including malalignment and instability [24]. Malalignment greater than 3° has been identified in up to one‐third of patients undergoing conventional TKA, potentially causing pain and instability [13], especially given that most authors recommend achieving a limb alignment within ±3°.

While predisposing factors for lower satisfaction have been identified, factors associated with better outcomes after TKA have also been recognized. These include older age, severe osteoarthritis, better baseline function, and realistic expectations. Conversely, factors associated with lower satisfaction include impaired mental health, poor baseline physical health, and postoperative complications [5, 6, 12].

With advances in perioperative management, implant design, and surgical techniques, there is growing optimism regarding improvements in patient satisfaction. The use of modern instrumentation and implants allows for better replication of the native knee anatomy and biomechanics. However, achieving and confirming precise overall alignment and proper balancing of flexion and extension spaces intraoperatively can be challenging with traditional manual, guide‐based instruments. Robotic assistance has proven effective in various areas, achieving precision of up to 0.05 mm [15]. Additionally, properly managing patient expectations before and after TKA can help avoid frustrations stemming from unrealistic expectations [18].

Published studies have compared early outcomes between robotic‐assisted total knee arthroplasty (RA‐TKA) and conventional manual TKA. Results indicate that RA‐TKA is associated with reduced pain, better early functional recovery, and shorter hospital stays compared to conventional TKA [23, 27].

The objective of our study is to evaluate and quantify the level of patient satisfaction and functional outcomes after robotic‐assisted TKA.

## METHODS

A prospective review was conducted on a cohort of patients who underwent unilateral RA‐TKA (Mako; Stryker) between November 2018 and December 2020. Ethical review was undertaken by the Health and Disability Ethics Committee of our Center. Informed consent was taken from all patients for use of data. No funding was required for this study.

Inclusion criteria consisted of patients undergoing primary RA‐TKA for osteoarthritis with a minimum follow‐up of 3 years. Patients who lacked postoperative satisfaction data regarding TKA or did not have complete required information were excluded.

### RA‐TKA operative technique

All patients underwent general anaesthesia (combining inhalation and intravenous methods). A tourniquet was used to create a bloodless surgical field. The approach was via a medial parapatellar incision, with careful handling of soft tissue release. Special attention was given to the anteromedial tibial soft tissue release to avoid injuring the patellar tendon.

The robotic system (MAKO®; Stryker) was calibrated and configured following a standardized protocol before surgery. Preoperative computed tomography (CT) scans of the hip, knee, and ankle were obtained using the TKA application platform to create a patient‐specific three‐dimensional model. This data was uploaded to the robotic system to plan the implant sizes preoperatively.

Intraoperatively, guides for the robotic system's antennas were fixed on both the tibia and femur, and registration was performed by capturing 40 structured points on the bony surface of each bone. During the registration of the 40 points on the bone surface, a precision level of less than 0.5 mm was desired to proceed with the procedure. All measurements were performed intraoperatively using the robotic system's user interface, which has a resolution of 0.5 mm for distances and 0.1° for angles.

The goal was to restore the pre‐arthritic medial proximal tibial angle (MPTA) within a safe range of 84° (varus) to 92° (valgus), which represents the knee's native alignment. The tibial slope was set to match the native medial tibial inclination. On the femoral side, the femoral component was positioned to restore the height of the medial joint line in both extension and flexion. Flexion and extension spaces were balanced by adjusting the distal lateral and posterior lateral femoral resection levels. Flexion space: The target was 1–2 mm of residual laxity in the medial compartment and 1–3 mm of residual laxity in the lateral compartment. Extension space: The goal was 1–2 mm of residual laxity in both compartments, ensuring the hip‐knee‐ankle (HKA) angle remained within a safe range.

The tibial and femoral resections were performed according to the intraoperative plan defined by the surgeon using the robotic system with haptic feedback assistance. Since a CT‐based navigation tool was used, all planned resection thicknesses were of bone, excluding cartilage. The robotic system allowed the surgeon to move the oscillating saw within the defined cutting plane and within haptic boundaries, thereby protecting soft tissues. The patella was routinely resurfaced using a conventional oscillating saw, with an asymmetric patellar implant installed. Regarding the implants, all patients received a PS implant with posterior cruciate ligament (PCL) sacrifice.

Patient rehabilitation was conducted by physical therapists at our centre following a standardized protocol. This included immediate postoperative rehabilitation, starting with the use of a continuous passive motion machine from recovery, direct mobilization, and immediate full weight bearing with the aid of crutches. Under the supervision of the physical therapist, patients were encouraged to perform active flexion and extension exercises from Day 1. On average, patients stayed at our facility for two nights. After this, patients followed a rehabilitation program appropriate to the surgery.

The use of orthopaedic canes was recommended for at least the first 2 weeks. All patients received routine prophylaxis with oral anticoagulation for 3 weeks following surgery. The first postoperative appointment at the outpatient clinic occurred at two weeks. Clinical follow‐up was conducted at 2 weeks, 4 weeks, 2 months, 3 months, 6 months, and 1 year postoperatively.

### Data extraction

**Figure 1:**
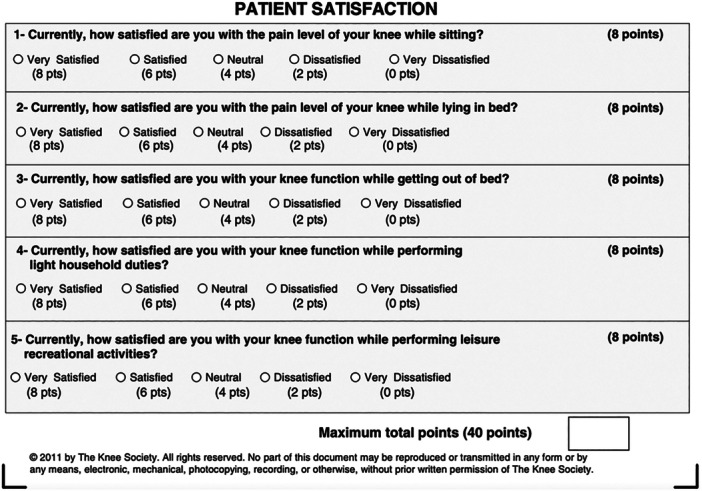
The patient satisfaction section of the 2011 KSS scoring system. KSS, Knee Society Score.

**Figure 2:**
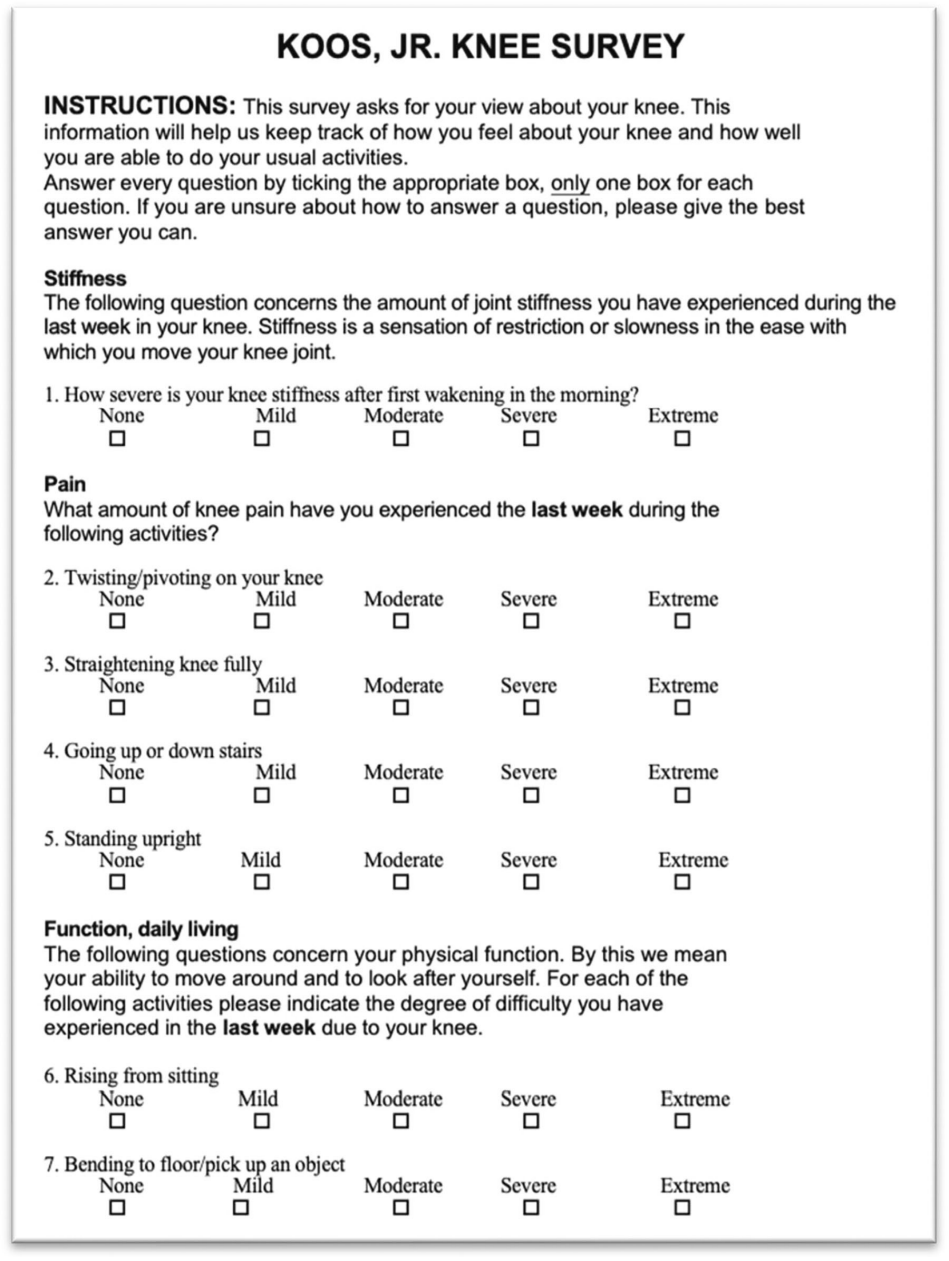
Knee Injury and Osteoarthritis Outcomes Score for Joint Replacement (KOOS JR).

Demographic data, 12‐month postoperative satisfaction (2011 KSS Satisfaction Survey) (see Figure 1) [25], and the Knee Injury and Osteoarthritis Outcome Score Junior (KOOS JR) [20] (see Figure 2) were collected preoperatively and at 12 months postoperatively. Demographic data included age, sex, body mass index (BMI), and postoperative complications. Comorbidities assessed included hypertension, dyslipidemia, arthritis, cardiac diseases, respiratory conditions, depression, and anxiety.

Patients were provided with the Knee Society Score (KSS) Satisfaction Survey and the KOOS JR, which they were required to complete independently at 1 year postoperatively.

### KSS 2011 Satisfaction Survey

In the KSS 2011 Satisfaction Survey [25], which has a maximum score of 40 points to evaluate overall satisfaction, patients are generally considered satisfied if they achieve a score of 30 points or higher. This corresponds to approximately 75% of the total score and reflects a positive perception of the surgical outcome in terms of overall satisfaction (see Figure 1).

### KOOS JR

Clinical outcomes were evaluated using the KOOS JR [20], a shortened version of the KOOS [26] that assesses patient stiffness (one item), pain (four items), and daily living functions (two items). Scores range from 0 to 100, where a score of 0 indicates total knee disability and 100 indicates perfect joint health. This is a joint‐specific, patient‐reported score designed to evaluate outcomes following TKA (see Figure 2).

### Data analysis

Predictors of postoperative dissatisfaction (Satisfaction ≥30 points) were estimated, adjusted for age, sex, and BMI. For the bivariate analysis, categorical variables were analysed using the *χ* ^2^ test. Quantitative variables were analysed using the Student's *t*‐test for normal distribution and the Mann–Whitney *U* test for non‐normal distribution.

A multiple logistic regression model was configured for postoperative dissatisfaction and the presence of severe‐extreme scores in the preoperative KOOS JR survey, adjusting for sex, age (continuous), and BMI (continuous). Results are presented as adjusted odds ratios (OR) by sex and age, with 95% confidence intervals (CI). The significance level was defined as *p* < 0.05. All statistical analyses were performed using STATA version 18.5.

## RESULTS

A total of 310 patients underwent unilateral RA‐TKA between November 2018 and December 2020. All patients were contacted between September and December 2024 for a review. At follow‐up, 270 patients were available, giving a follow‐up rate of 87% at a mean follow‐up time of 36 months after surgery (standard deviation [SD]: 5.4, range: 19.8–52.2 months). Of these, 58.2% (*n* = 157) were female, with a mean age of 70 years (SD: 8.2) and a BMI of 28.1 (SD: 4.2). Of the total number of patients, 72% had a varus deformity (194 patients), while the remaining patients presented with a valgus deformity.

The mean preoperative KOOS JR score was 45.9 (SD: 10.7), while the postoperative KOOS JR score was 78.2 (SD: 12.1), with an average total improvement of 32.2 points (SD: 16.1) (see Table 1).

**Table 1:** Univariate analysis (*N* = 270).

Characteristics	Mean (SD)
Female sex, %	157 (58.2)
Age	70.0 (8.2)
BMI	28.1 (4.2)
Preoperative KOOS	45.9 (10.7)
Postoperative KOOS	78.2 (12.1)
Difference in pre‐post points	32.2 (16.1)
Satisfaction points	34.56 (6.56)
Dissatisfaction <30 points, %	8 (7.3)

A total of 92.6% (*n* = 250) of patients scored 30 points or more on the satisfaction survey, placing them in the group classified as satisfied or very satisfied with the intervention. Meanwhile, 7.4% (*n* = 20) scored less than 30 points, indicating dissatisfaction with the surgery. No significant differences were observed between the groups in terms of sex, age, BMI, preoperative deformity or preoperative KOOS JR scores. However, patients in the satisfied group had significantly higher postoperative KOOS JR scores (*p* = 0.0001) (see Table 2).

**Table 2:** Bivariate analysis satisfaction/dissatisfaction (<30 points).

	Satisfaction	Dissatisfaction	
Characteristics	*n* = 250 (92.6%)	*n* = 20 (7.4%)	*p* value
Female sex, *n* (%)	142 (58.9)	15 (75.0)	0.31
Age, mean (SD)	69.9 (8.1)	71.9 (9.4)	0.5
BMI, mean (SD)	28.2 (4.1)	27.1 (5.1)	0.48
Preoperative KOOS, mean (SD)	46.3 (10.8)	40.4 (7.8)	0.12
Postoperative KOOS, mean (SD)	79.4 (11.6)	62.4 (2.4)	0.0001
Pre‐post difference, mean (SD)	33.0 (16.3)	22.0 (8.2)	0.06

**Figure 3:**
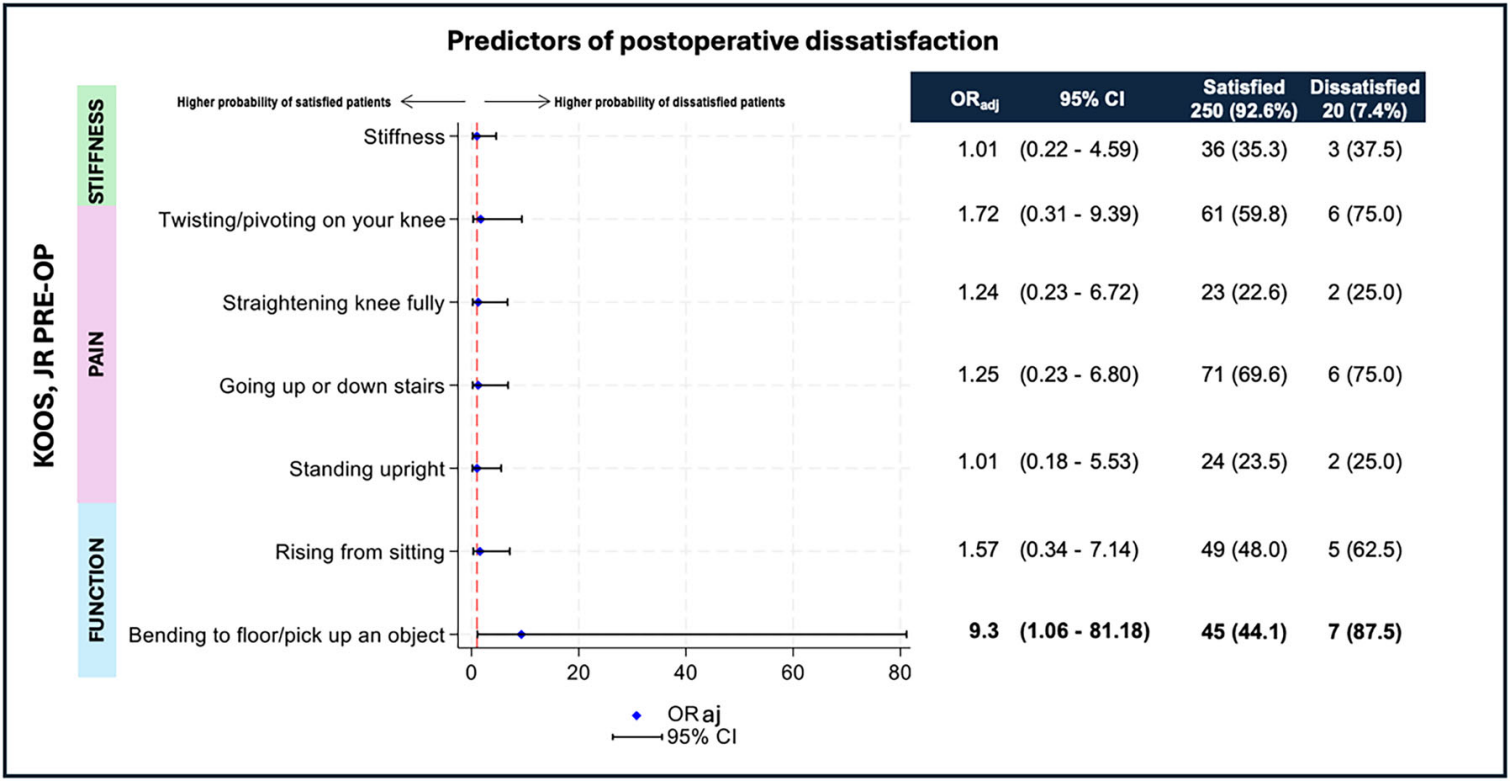
Predictors of dissatisfaction based on change points in preoperative KOOS JR. KOOS JR, Knee Injury and Osteoarthritis Outcome Score for Joint Replacement. adj, adjusted analysis; CI, confidence interval; OR, odds ratio; PRE‐OP, preoperstive.

When the preoperative KOOS JR was analysed by individual items, patients who reported severe or extreme difficulty in picking up an object preoperatively had a higher likelihood of postoperative dissatisfaction (OR: 8.86; 95% CI: 1.05–74.7). These results remained consistent after adjusting for sex, age, and BMI (OR: 9.3; 95% CI: 1.06–81.18) (see Figure 3).

In the present study, the following complications were noted among patients who underwent RA‐TKAs: (a) deep vein thrombosis (14/270, 5.18%); (b) periprosthetic fracture (2/270, 0.74%); (c) pulmonary embolism (2/270 TKAs, 0.74%). The 14 cases of deep vein thrombosis and the two cases of pulmonary embolism received anticoagulation therapy in the form of low‐molecular‐weight heparin. Although some patients cited the development of operative complications as a reason for dissatisfaction, it was not found to be significantly correlated with lower patient satisfaction (*p* = 0.174). No knee had a pin site fracture, pin tract infection, patellar dislocation, patellar fracture or peroneal nerve palsy.

## DISCUSSION

The primary finding of this study was a high postoperative satisfaction rate of 92.6%, accompanied by a substantial improvement in functional outcomes as measured by KOOS JR scores at 12‐month follow‐up. These results are in line with recent literature, which reports satisfaction rates of approximately 85–90% following TKA [8, 11, 19, 32]. Although all procedures in our study were performed using robotic assistance, the absence of a conventional (non‐robotic) control group prevents us from attributing these favourable outcomes solely to the robotic technology.

Robotic systems have been designed to enhance surgical precision and consistency, particularly in achieving optimal alignment and ligament balancing [2, 16]. Preoperative planning and real‐time intraoperative feedback may contribute to more accurate implant positioning and improved outcomes [9, 22, 29], including prosthesis survivorship [4, 5, 17, 28]. While these technological advantages are promising, we did not directly evaluate their impact in this study, and no direct comparisons were made with conventional surgical techniques. Therefore, any potential benefit of robotic assistance should be interpreted with caution and not assumed as the primary driver of patient satisfaction.

The concept of patient satisfaction in TKA has evolved beyond traditional metrics such as implant survivorship and range of motion. Increasingly, patient‐reported outcomes are recognized as essential indicators of success. Previous research has demonstrated discrepancies between surgeon‐assessed and patient‐reported outcomes, highlighting the need to incorporate the patient's perspective into surgical evaluation. Patient satisfaction is influenced by both internal factors—such as age, psychological state, and expectations—and external factors, including hospital environment and surgical technique [1, 10, 31]. Independent factors can be managed by the surgeon. However, it is unclear which patient‐specific factors, such as mental health status and expectations, may influence outcomes and satisfaction after TKA. To assess mental health status, there are internationally validated scales, including the Hospital Anxiety and Depression Scale (HADS), which has also been validated in Spanish [33]. On the other hand, pain and functional limitations are among the primary causes of dissatisfaction. Therefore, it is essential to objectively measure outcomes using instruments validated in the literature and in our language, such as the Knee Injury and Osteoarthritis Outcome Score (KOOS) [7, 30].

While numerous studies have confirmed that precise ligament balancing, appropriate flexion and extension gaps, and accurate limb alignment are critical for the longevity of a TKA, achieving these objectives in conventional TKA may still fall short. RA‐TKA represents a significant advancement in the surgical management of severe knee osteoarthritis, noted for its high precision and potential to improve clinical outcomes [9, 22, 27, 29]. Despite these advancements, a 7.4% dissatisfaction rate highlights the importance of addressing factors that contribute to unmet patient expectations or residual functional limitations.

A second finding of this analysis is the correlation between KOOS JR scores and a higher likelihood of postoperative dissatisfaction. While preoperative KOOS JR scores did not differ significantly between satisfied and dissatisfied groups, postoperative KOOS JR scores were significantly higher (*p* = 0.0001) among satisfied patients. This underscores the critical role of postoperative functional improvement in determining satisfaction. The significant correlation between higher postoperative KOOS JR scores and satisfaction suggests this metric is a reliable indicator of surgical success. Functional recovery, as measured by KOOS JR, appears to play a larger role in determining satisfaction than demographic variables such as sex, age, or BMI. The analysis found no significant differences in terms of overall preoperative KOOS JR scores between satisfied and dissatisfied patients, except in cases where extreme difficulties were reported in specific activities, such as reaching for an object. Patients reporting severe or extreme difficulty in picking up objects preoperatively were significantly more likely to experience dissatisfaction postoperatively (OR: 9.3 after adjustment). This finding suggests that specific functional deficits may serve as predictors of suboptimal outcomes, potentially reflecting underlying biomechanical or psychological factors that persist despite surgical intervention.

The complication rate in the present study was 6.66%, and the complications encountered included thrombotic events and periprosthetic fracture. Various studies have correlated complications with low levels of patient satisfaction [12, 21, 32]. However, the present study found that patient satisfaction was not significantly influenced by the development of operative complications (*p* = 0.174).

A potential technical limitation in our protocol was the routine resurfacing of the patella using a conventional oscillating saw and an asymmetric patellar implant. The MAKO system does not include segmentation of the patella, making it difficult to precisely control the final anterior offset. Variability in anterior offset could affect patellofemoral biomechanics and, consequently, patient‐reported outcomes. In this context, resurfacing all patellae may introduce heterogeneity into the surgical results. Surgeons must be cautious to avoid overstuffing the joint by accounting for the original patellar thickness. The lack of robotic control in patellar preparation may lead to inconsistencies that could impact functional recovery and satisfaction.

Finally, since our study lacked a control cohort of patients who underwent conventional TKA, we cannot conclude that the high satisfaction rates observed were directly attributable to robotic assistance. Further research—including randomized studies or matched‐cohort analyses comparing robotic and conventional techniques—is necessary to isolate the specific contributions of robotic technology. Additionally, future studies should aim to expand the sample size and explore strategies for addressing preoperative functional deficits that may predispose patients to dissatisfaction.

## CONCLUSION

In summary, our findings demonstrate high levels of patient satisfaction and significant functional improvement following TKA performed with robotic assistance. Nonetheless, due to the absence of a control cohort, we cannot assert that robotic assistance was the sole or primary factor responsible for these outcomes. However, factors such as severe difficulties in preoperative KOOS JR scores may predict postoperative dissatisfaction. These findings highlight the importance of comprehensive preoperative evaluation to optimize outcomes and patient satisfaction.

## AUTHOR CONTRIBUTIONS

All the authors contributed to the design, analyses and reporting for this manuscript. Both authors read and approved the final submitted manuscript.

## CONFLICT OF INTEREST STATEMENT

The authors declare no conflicts of interest.

## ETHICS STATEMENT

Informed consent was taken from all patients for the use of data.

## Supporting information

Supporting material.

## Data Availability

Data are available on request from the authors.
