# Supplementary material for: High satisfaction and functional improvement following robotic‐assisted total knee arthroplasty: A Latin American cohort study
Source: J Exp Orthop. 2025 Jul 7;12(3):e70344. doi: 10.1002/jeo2.70344 (PMC12231059; doi:10.1002/jeo2.70344)
Supplement: Supplementary file 1 — Supporting material. [file JEO2-12-e70344-s001.docx]

This study evaluated patient satisfaction and functional outcomes after robotic-assisted total knee arthroplasty (RA-TKA) in a Latin American cohort. Among 270 patients with 3-year follow-up, 92.6% reported satisfaction and showed significant improvement in KOOS-JR scores. Postoperative function strongly correlated with satisfaction. RA-TKA demonstrated excellent outcomes, although further studies are needed to isolate the impact of robotic technology.


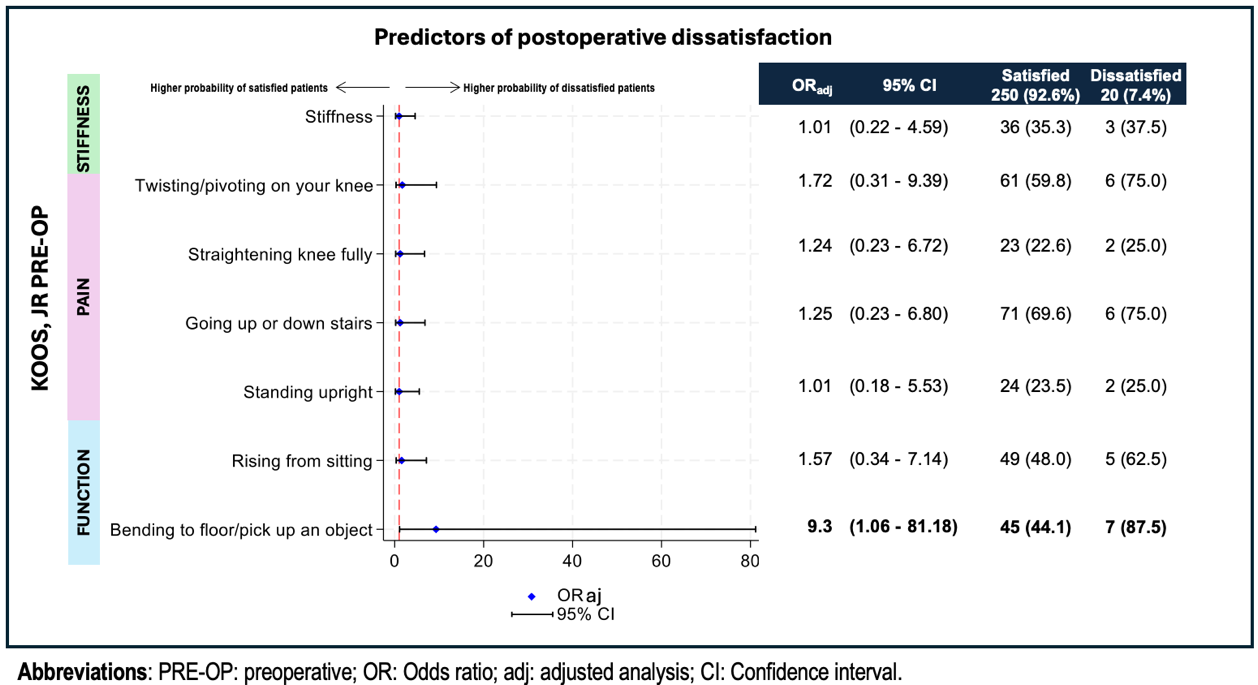


**Supplementary Figure.** Predictors of dissatisfaction based on change points in preoperative KOOS JR.
